# Body Composition and Physical Fitness in Five-a-Side Soccer for the Blind Players: A Penalized Regression Approach

**DOI:** 10.3390/sports14100447

**Published:** 2026-10-09

**Authors:** Boryi A. Becerra-Patiño, Juan David Paucar-Uribe, Miguel Andrés Peñuela-Argüello, Cristián David Zapatá-Piratova, Armando Monterrosa-Quintero, Rodrigo Yáñez-Sepúlveda, José Francisco López-Gil, José Pino-Ortega

**Affiliations:** 1 Programa de Doctorado en Ciencias de la Actividad Física y del Deporte, Faculty of Sport Science, University of Murcia, 30720 San Javier, Spain; boryialexander.becerrap@um.es or babecerrap@pedagogica.edu.co; 2 Faculty of Physical Education, National Pedagogical University, Bogotá 480100, Colombia; jdpaucaru@upn.edu.co (J.D.P.-U.); mapenuelaa@upn.edu.co (M.A.P.-A.); cdzapatap@upn.edu.co (C.D.Z.-P.); 3 Altius Performance Laboratory, Physical Education and Sports Program, Universidad Surcolombiana, Neiva 410001, Colombia; adomonterrosa@gmail.com; 4 Faculty of Education and Humanities, School of Sport Sciences, Universidad Andres Bello, Viña del Mar 2520000, Chile; rodrigo.yanez.s@unab.cl; 5 Department of Sport Sciences, Faculty of Sport and Health Sciences, Fit Generation Research Institute, AD500 Andorra la Vella, Andorra; 6 School of Medicine, Universidad Espíritu Santo, Samborondón 092301, Ecuador; 7 Vicerrectoría de Investigación y Postgrado, Universidad de Los Lagos, Osorno 5290000, Chile; 8 Faculty of Sport Science, University of Murcia, 30100 Murcia, Spain; josepinoortega@um.es

**Keywords:** visual impairment, athletic performance, physical fitness, somatotype, penalized regression, paralympic sport, Elastic Net

## Abstract

**Background**: Five-a-side soccer for the blind (F5) is a Paralympic sport in which there are various performance variables, including anthropometric factors and physical fitness. However, despite this recognition, there is little scientific literature on performance analysis using algorithms to identify the main variables in this sport. **Objective**: The objectives of this study were (i) to identify key performance indicators in F5 players based on body composition and physical fitness variables and (ii) to rank anthropometric and physical performance variables using machine learning (ML) algorithms. **Materials and Methods**: A cross-sectional study was conducted to characterize the body composition and physical fitness levels of F5 players. A total of 20 F5 players participated. To address the high dimensionality and multicollinearity of the anthropometric variables while mitigating the risk of overfitting, penalized regression models (Elastic Net and Lasso algorithms) were implemented. **Results**: While unadjusted bivariate correlations suggested associations between body composition and performance, none survived False Discovery Rate (FDR) correction (pFDR ≥ 0.05). However, regularized multivariate models isolated exploratory candidate variables. Cross-validated performance was null (R^2^cv = 0.00%) for countermovement jump (CMJ) jump height, CMJ power, COD 5-0-5 and 20 m sprint, and moderate only for speed 0–5 m (R^2^cv = 59.42%; RMSE_cv_ = 0.08 s). **Conclusions**: No statistically significant differences in anthropometric, body composition, or physical fitness profiles were detected between attackers and defenders (*p* > 0.05). Penalized ML algorithms identified a small set of exploratory candidate variables associated with specific physical tasks. Due to the small sample size (n = 20), these models should be considered exploratory and hypothesis-generating.

## 1. Introduction

Five-a-side soccer for the blind (F5) is a Paralympic sport played by people with visual impairments [1] that was first contested at the 2004 Paralympic Games in Athens. It is characterized be the use of a sound-emitting ball that allows players to process information through other senses, such as hearing and kinesthesia [2,3]. The game is played on a field measuring 40 m long by 20 m wide, separated by side kickboards designed to increase the pace of play and encourage players to use these lateral boundaries for orientation. Four field players participated, with only blind 1 (B1) players (totally blind) eligible as field players [4], along with a goalkeeper without visual impairment. To ensure a fair competition, the four field players wear blindfolds. In addition, the players receive instructions and suggestions from three guides: the goalkeeper, the coach standing on the sideline, and a guide positioned behind the offensive goal where they must attempt to score [5].

F5 is characterized as an intermittent sport in which athletic performance is determined by body composition [6,7] and physical fitness [8,9] variables, particularly because these variables allow players to maintain the intensity of their actions throughout the entire game [10]. A systematic review on body composition and anthropometric factors in F5 indicates that the somatotype of F5 players tends to be mesoendomorphic and that variables such as muscle mass, fat mass, and body weight may influence athletic performance, although it is not conclusive whether this can be generalized to different contexts and in relation to playing position [11]. Therefore, further studies are needed to establish relationships between body composition variables and to identify how these relate to physical fitness variables in F5 players from different contexts and competitive levels [12]. According to our information, few studies have been conducted in the international context that use machine learning (ML)-based penalized regression models to identify the essential morphological explanatory factors for each performance metric evaluated in F5.

Furthermore, the study of physical variables is commonly linked to the assessment of strength, since players must adapt to specific competitive demands such as accelerations, decelerations, physical struggles, and battles for the ball [13,14]. Changes of direction allow players to perform defensive, offensive, and transitional actions, and speed appears to be a key factor when dribbling the ball [15]. Physical performance in F5 can be defined as a complex, dynamic, and multifactorial process in which players need to integrate different processes to apply force in offensive actions such as accelerating and shooting, changes of direction to maintain the intensity of the game, and speed to cover distances in short periods of time [16,17].

The physical demands of F5 generate specific aerobic and anaerobic responses that players must stimulate to maintain athletic performance throughout the game and delay fatigue [18]. Likewise, competitive demands are key determinants of performance. Some research on F5 has shown that the outcome is a crucial indicator, as losing teams tend to exhibit higher physical workloads [13,14]. Consequently, it is important to continue characterizing how F5 players exhibit body composition and physical fitness variables to determine how this influences player performance and, thereby, to develop increasingly specific training programs [19]. Using studies that have analyzed body composition and physical fitness in F5 players, these variables have typically been analyzed independently; as a result, to date, few studies have been reported that attempt to determine the profile of F5 players using body composition and physical fitness variables [20] through the use of ML. Accordingly, the objectives of this study were (i) to identify key performance indicators in F5 players based on body composition and physical fitness variables and (ii) to rank anthropometric and physical performance variables using ML algorithms.

## 2. Materials and Methods

### 2.1. Design

A cross-sectional study [21] was conducted to characterize the body composition and physical fitness levels of F5 players.

### 2.2. Participants

A study was conducted with 20 F5 players, including 11 attackers and 9 defenders. The selected players were field players; therefore, goalkeepers were not included. Convenience sampling was used because the group had already been selected based on the national team coach’s criteria. The inclusion criteria were as follows: (i) being a member of the national team; (ii) having played in at least one national tournament; (iii) training at least 4 times a week; (iv) completing all tests. The exclusion criteria were as follows: (i) having medical conditions that hinder physical exertion; (ii) recovering from an injury or undergoing rehabilitation; (iii) training with the national team without being registered for a national tournament. All the athletes evaluated were men; they had a functional classification of B1, and their athletic experience was 8 years for the attackers and 9 years for the defenders.

### 2.3. Instruments, Variables Assessed, and Assessment Procedure

Before taking the various tests, the athletes participated in two familiarization sessions for each test. During these sessions, the purpose of each test was first explained to them, and their safety was ensured through audible signals that indicated the start and end of each test. Each athlete made three attempts at each of the physical fitness tests, and the best result was recorded. The assessment was conducted in two phases: one to evaluate body composition and another to evaluate physical fitness.

Stage 1. Before conducting the evaluations, all athletes and the coaching staff were gathered for a brief explanation of the procedure. All evaluations were conducted at the national team’s facilities. The first step was to ask the athletes to be barefoot and wear athletic clothing. Total body mass was measured using an OMRON scale (model HBF-514C, Kyoto, Japan), which has an accuracy of 0.1 kg using bioelectrical impedance, and the players’ height was measured according to the Frankfurt standard using a portable stadiometer with an accuracy of 0.1 cm (Seca, 213, Hamburg, Germany), following the standard procedures described in another study [22]. Two International Society for the Advancement of Kinanthropometry (ISAK) certified evaluators were responsible for conducting the body composition assessment. The reliability of the assessments was ensured through duplicate measurements, resulting in a technical error of measurement (TEM) of less than 2.5%.

Stage 2. Each of the physical fitness tests was administered on the national team’s training field and conducted in the following order: countermovement jump (CMJ) [23,24], 5-0-5 changes of direction [25], and 0-to-20 m sprint test [26]. Data were collected using the My Jump Lab app for jumps (with r values > 0.91, *p* < 0.05), COD-Timer 5-0-5 for changes of direction (r = 0.96; intraclass correlation coefficient (ICC = 0.97)), and Runmatic for speed (r = 0.94–0.99, *p* < 0.001). Each assessment was conducted using an iPhone 14 model 2022 (Apple, Cupertino, CA, USA). Additionally, cones and markers were used to delineate the test areas. If an attempt was invalid, it was repeated after 10 min to ensure the athlete had fully recovered. The schedule for conducting the physical fitness tests on the playing field was set for two days. On the first day, strength tests were performed using the vertical jump (CMJ) to obtain measurements of force (N), power (W), jump height (cm), flight time (ms), and speed (m/s). The CMJ assessments were conducted from a frontal view, with the mobile device positioned 3 m away and recording at 240 fps from a height of less than 1.0 m. On the second day, the COD-Timer 5-0-5 test and the speed test were conducted, with a minimum of 3 min of recovery time between each test. For the COD-Timer 5-0-5 test, the device was positioned 5 m away, and for the speed test, it was positioned 10 m away. The goal was to conduct the assessments every 48 h to facilitate the athletes’ recovery.

Each field test was always conducted at the same time (from 4:00 p.m. to 6:00 p.m.) under similar environmental conditions (average temperature: 13.1 °C; humidity: 77–83%). Before beginning, the F5 players followed a standardized warm-up protocol. Running exercises from the FIFA 11+ manual were performed continuously for 40 s to 1 min. The first exercise consisted of running in a straight line within a designated space at moderate intensity. Next, the players ran in a straight line, moving their hips outward and inward with each step to activate the lateral and internal hip muscles and improve lateral mobility. Next, the players ran in circles around a teammate, alternating directions. Afterward, they ran in a straight line, jumping upon contacting a teammate at a certain distance. Finally, they performed quick sprints in a straight line, alternating directions between attackers and defenders [27].

#### 2.3.1. CMJ

The CMJ vertical jump is performed from a standing position with hands on the hips, in which the athlete must bend the knees to a 90° angle and then jump using the momentum provided by the stored energy [28]. This test is evaluated based on a single jump, performed in the shortest time possible while aiming to achieve the greatest possible height. The variables assessed were force (N), power (W), jump height (cm), flight time (ms), and velocity (m/s). To facilitate the jump, a 1 m^2^ rectangle was marked off with cones, which the athlete acknowledged by touching with their hands before jumping.

#### 2.3.2. COD 5-0-5

Changes in direction were assessed using the COD-Timer 5-0-5 to evaluate changes in direction over a 15 m course at the highest possible intensity, with a 180° turn generating accelerations and decelerations in the shortest possible time. The variables assessed were time (s), average speed (km/h), contact time (ms), and COD deficit (s).

#### 2.3.3. 5, 10, 15, and 20 m Sprints

The test is performed in a single run covering 5, 10, 15, and 20 m in a straight line, completed as quickly as possible while considering each of the phases: From 0 to 5 m, it measures acceleration over a short distance. From 5 to 10 m, it consists of maintaining speed with momentum determined by the acceleration over the first few meters. From 10 to 15 m, it evaluates speed over a medium distance, and finally, from 15 to 20 m, it measures speed over the longest distance of the test, yielding the test’s maximum values. The speed assessment was conducted with two guides: one at the start of the test and another at the finish, who clapped when the participant crossed the 20 m mark so they could slow down and avoid tripping.

### 2.4. Ethical Procedures

Each of the procedures complied with the ethical guidelines and recommendations of the Declaration of Helsinki [29] and with the principles established by Resolution 8430 of the Colombian Ministry of Health, which classified the study as low risk according to Colombian regulations based on standards and guidelines for research involving noninvasive procedures in human subjects [30]. The study was approved by the Ethics Committee of the University of Murcia (ID: M10/2025/572). Initially, the national team’s head coach was contacted to inform him about the study proposal. Each player signed an informed consent form. Afterward, a date was set for the evaluations. Before the tests, the researchers conducted several familiarization sessions to help the players become acquainted with the tests and the high level of monitoring involved, thereby reducing the likelihood of errors during the evaluation. The players were scheduled to arrive at their regular training times and were grouped in pairs for the body composition and physical fitness assessments. At the end of each assessment, the results were exported to an Excel file, and the data were organized into a spreadsheet containing each player’s results. Each player was assigned a pseudonym to ensure anonymity. Finally, a database was created for subsequent statistical analysis.

### 2.5. Statistical Analysis

Statistical analyses and ML modeling were conducted in the RStudio environment (version 2025.09.2 Build 418; R Core Team). Following the verification of normality (Shapiro–Wilk) and homoscedasticity (Levene’s test), parametric continuous variables were reported as mean ± standard deviation (SD), whereas non-parametric data were expressed as median [interquartile range, IQR]. Between-group comparisons across tactical positions (attackers vs. defenders) were evaluated using independent-samples Student’s *t*-test, Mann–Whitney U test, or Welch’s *t*-test when heteroscedasticity was detected to prevent the inflation of type I error rates. Given the absence of statistically significant differences between tactical positions (*p* > 0.05), data (n = 20) were pooled for subsequent exploratory regularized analyses via Spearman’s rank correlation coefficients (rho), with effect magnitudes classified according to Hopkins’ criteria [31]. To control the False Discovery Rate (FDR) arising from simultaneous correlation testing, *p*-values were adjusted using the Benjamini–Hochberg procedure. To address the high dimensionality and severe multicollinearity inherent to the 32 anthropometric and body composition indicators relative to the sample size (n = 20), penalized linear regression models were implemented via the glmnet, caret, and boot packages in R. To avoid subjective pre-selection bias among collinear kinanthropometric indicators, the full predictor matrix was retained, relying on the intrinsic grouping effect of the Elastic Net penalty to handle correlated features. Prior to model training, all continuous independent predictors were internally centered and scaled (z-scores: mean = 0.0, SD = 1.0) within each fold to prevent penalization bias driven by differing measurement units; regression coefficients were subsequently back-transformed into their original physical units for field interpretability. To balance the L1 (Lasso) and L2 (Ridge) penalties, Elastic Net regression was applied [32] to sprint, change of direction, and jump height metrics, where the mixing hyperparameter α was tuned across a structured grid (α from 0.1 to 0.9 in 0.1 increments) using an internal fivefold cross-validation scheme (k = 5 folds, n = 4 observations per fold). For the Power CMJ model, a strictly parsimonious Lasso regression (α = 1.0) constrained to the top 5 candidate predictors was specified to prevent oversaturation given the cohort size. Regularization parameters (λ) were selected based on the conservative λ_1se_ criterion (the most parsimonious model within one standard error of the minimum cross-validation loss) via cv.glmnet, effectively shrinking redundant and non-informative coefficients to zero. Out-of-fold performance was evaluated using the cross-validated Root Mean Square Error (RMSE_cv_) and the cross-validated coefficient of determination (R^2^_cv_), defined as R^2^_cv_ = 1 − (MSE_cv_/Var(y)) using the sample mean as the baseline null model, with negative out-of-sample values truncated to 0.00%. To evaluate the stability of the selected features against sample perturbations in a small cohort, a non-parametric bootstrapping procedure (B = 500 iterations) was executed, tracking the selection frequency (the percentage of bootstrap resamples in which each predictor remained non-zero). Given the sample size (n = 20), these regularized analyses were treated as exploratory and hypothesis-generating rather than definitive predictive tools. Computational reproducibility was ensured across all analytical routines by fixing pseudo-random seeds (seed = 123). Statistical significance was set at *p* < 0.05. The complete, commented R workflow and dataset are available as a supporting information: https://doi.org/10.6084/m9.figshare.33260565 (accessed on 14 August 2026).

## 3. Results

This analysis details the morphophysiological and physical performance profiles of F5 players, segmented according to their tactical position (attackers versus defenders). To describe these characteristics, the empirical results have been structured into three tables encompassing basic anthropometric parameters, body composition fractioning, and lower limb power alongside other dynamic performance capacities (Table 1). The main findings of this study, based on hypothesis testing (Student’s *t*-test, Mann–Whitney U test, and Welch’s *t*-test), reveal that no statistically significant positional differences were detected between both positions in any of the analyzed variables. Based on these data, it could be hypothesized that the demands of the sport require similar physical development for all players, regardless of their role on the pitch.

This section describes tissue distribution using standard reference methods in kinanthropometry, such as Lee’s method for muscle mass estimation and the Kerr, Faulkner, and Carter protocols for adipose tissue. It is observed that muscle mass comprises between 37.53% and 38.41% of the total body mass, while body fat percentages (21.02% in defenders and 24.28% in attackers) and the sum of eight skinfolds (105.31 to 110.71 mm) maintain statistical parity. Based on these results, it is reasonable to hypothesize that morphological adaptations derived from training loads manifest uniformly across the entire squad, without an evident positional specialization (Table 2).

The evaluation of field physical capacities allows for the analysis of the lower limb power and the kinematic profile of these para-athletes. In the CMJ test, power output (1669.18 W to 1816.82 W) and jump height (31.47 to 32.35 cm) evidence a symmetric development of explosive leg strength for both groups. Furthermore, metrics for linear acceleration (20 m total sprint time in 3.30 to 3.56 s) and agility with change of direction (COD 5-0-5, with averages of 4.86 to 5.15 s) show no statistically significant differences. This lack of statistical differentiation suggests the hypothesis that actions involving rapid propulsion, jumping, and deceleration demand comparable integral capacities in both attackers and defenders, rather than requiring specialized mechanical profiles (Table 3). Given that the previous comparative analyses (Table 1, Table 2 and Table 3) demonstrated no significant differences between tactical positions (*p* > 0.05), data from the entire cohort were pooled to examine exploratory bivariate interactions.

**Figure 1:**
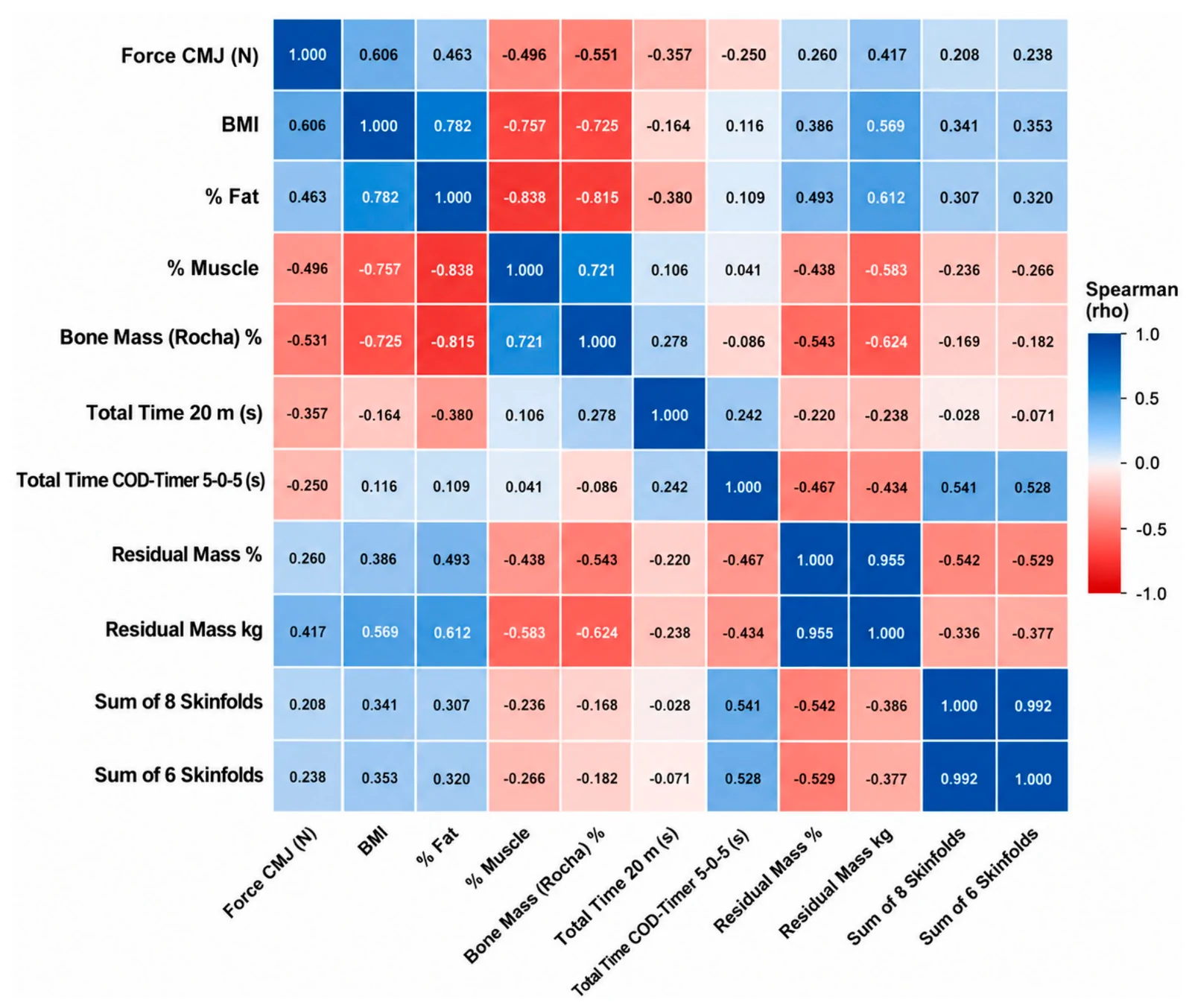
Spearman’s correlation analysis: interactions between body composition and physical performance in F5.

When evaluating the raw interactions between body composition and physical performance, the unadjusted Spearman’s heatmap (Figure 1) reveals initial exploratory trends.

**Figure 2:**
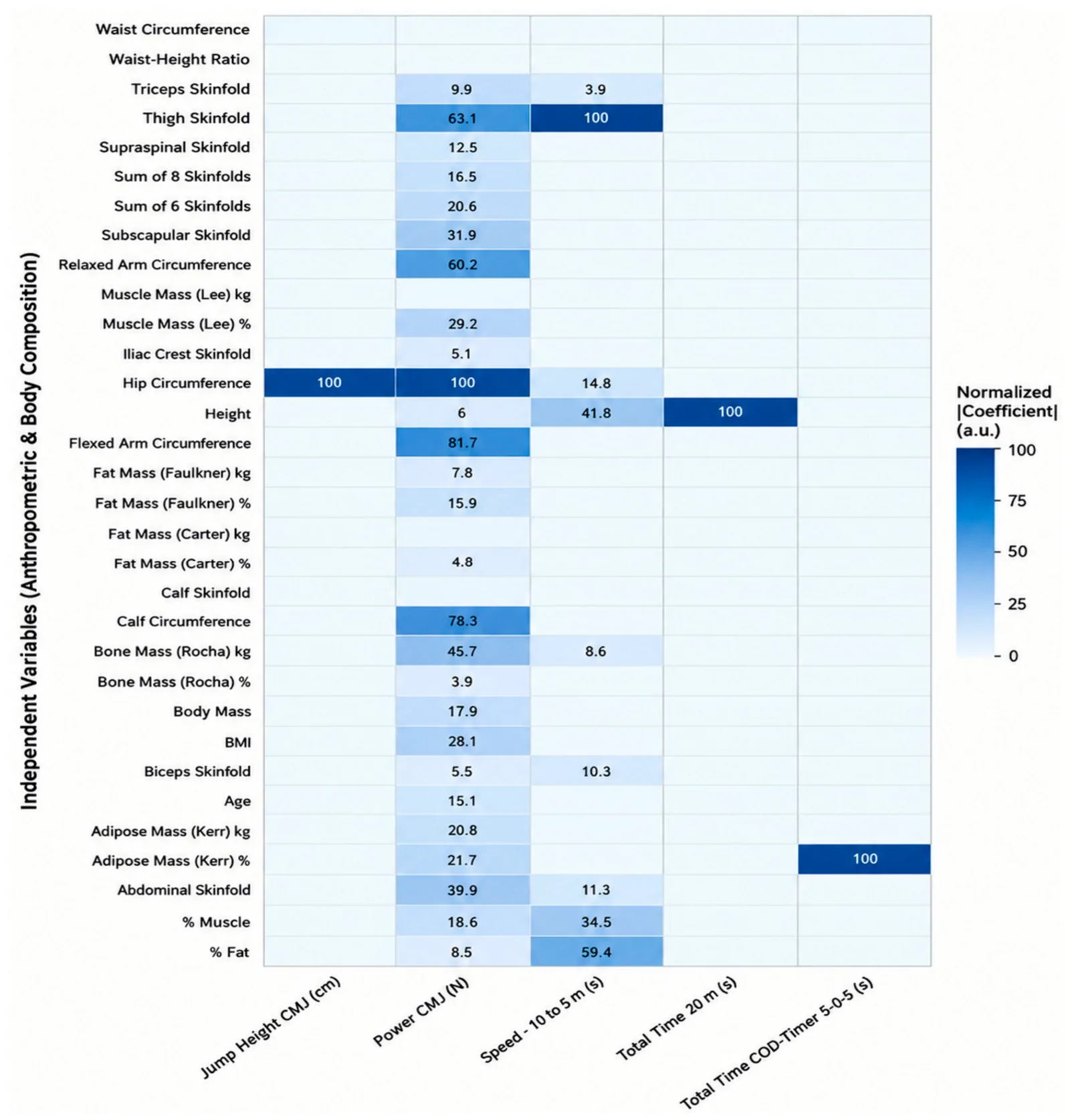
Consolidated candidate predictor importance matrix generated via penalized linear regression. The heatmap illustrates the normalized absolute standardized coefficient magnitude (|β|, rescaled from 0 to 100 a.u.) of anthropometric and body composition parameters across five distinct physical performance criteria. Values indicate relative regularized feature weighting rather than explained variance or percentage contribution, where darker shades represent higher candidate importance along the regularization path.

Before correcting for multiple comparisons, moderate-to-strong raw correlations were observed, such as absolute jump force (CMJ Force) correlating with body mass index (BMI) (*ρ* = 0.606, *p_unadj_* = 0.005) and bone mass percentage (*ρ* = −0.531, *p_unadj_ =* 0.017), as well as COD 5-0-5 time with the sum of eight skinfolds (*ρ* = 0.541, *p_unadj_* = 0.014). However, conducting multiple simultaneous bivariate tests on a small cohort (n = 20) severely inflates the risk of false positives (Type I error), these raw correlations must be interpreted solely as exploratory trends. First, the values correspond to standardized absolute coefficients; therefore, a value of 100 represents only the predictor with the highest coefficient within each dependent variable and does not imply an effect of magnitude 100 nor does it allow for a direct comparison of the importance of different performance tests. Second, by using the absolute values of the coefficients, the figure omits information about the direction of the association. Therefore, it is not possible to determine whether higher adiposity, muscle mass, circumference, or skinfold thickness is positively or negatively related to performance. Selection also depends on the penalty term (λ) and modeling decisions; thus, small changes in these parameters or in the sample could yield different selection patterns. For these reasons, the results should be interpreted as an exploratory representation of the relative importance of the predictors (Figure 2).

### 3.1. Variable Selection via Regularization

To address the severe multicollinearity and high dimensionality inherent to the 32 kinanthropometric indicators relative to the sample size (n = 20), regularized linear regression algorithms (Elastic Net and Lasso) were implemented. The regularization mechanism automatically contracted non-informative and redundant coefficients to zero under fivefold cross-validation. For Power CMJ, a strictly parsimonious Lasso model (α = 1.0) constrained to the top five candidate predictors was specified to prevent oversaturation in small-cohort modeling. All standardized coefficients derived from R (glmnet/caret) were mathematically transformed into unstandardized physical units using participant descriptive statistics (means and standard deviations). To verify the algebraic integrity of this conversion and ensure that no mathematical errors occurred during unstandardization, an arithmetic reconstruction example is provided for each equation. Because evaluating a linear regression model at the sample predictor means algebraically recovers the sample mean of the outcome, these worked examples serve strictly as an arithmetic check rather than evidence of predictive validity. Given the cross-sectional design and limited sample size, these equations represent exploratory morphological profiles specific to this national team rather than validated out-of-sample predictive tools.

### 3.2. Morphological Characteristics Associated with Vertical Jumping Performance

For CMJ jump height, Elastic Net regularization simplified the multi-feature matrix by retaining hip circumference as the sole exploratory candidate predictor:

Jump Height CMJ (cm) = 33.2732 − 0.0158 × (Hip Circumference, cm)(1)

Worked Example (Arithmetic Reconstruction): For an athlete exhibiting the cohort’s mean hip circumference (89.02 cm), the reconstructed jump height is 33.2732 − (0.0158 × 89.02) = 31.87 cm, which arithmetically reproduces the observed cohort sample mean of 31.87 cm. This calculation confirms the algebraic consistency of the unstandardization process but does not represent out-of-sample predictive validation.

For absolute CMJ power output, the strictly parsimonious Lasso model retained overall body size markers (BMI), lower-limb muscular perimeter (calf circumference), upper-body perimeter (flexed arm circumference), and muscle mass percentage:

Power CMJ (W) = 768.5968 + 2.3271 × (Body Mass, kg) + 16.2537 × (BMI, kg/m^2^) − 7.8498 × (Flexed Arm Circumference, cm) + 18.4556 × (Calf Circumference, cm) − 0.2193 × (Muscle Mass [Lee], %) (2)

Worked Example (Arithmetic Reconstruction): Inserting cohort mean values (body mass: 70.06 kg; BMI: 24.84 kg/m^2^; flexed arm circumference: 28.89 cm; calf circumference: 35.24 cm; muscle mass [Lee]: 39.17%) yields: 768.5968 + (2.3271 × 70.06) + (16.2537 × 24.84) − (7.8498 × 28.89) + (18.4556 × 35.24) − (0.2193 × 39.17) = 1750.38 W, which arithmetically reproduces the observed cohort sample mean of 1750.38 W (1750.38 ± 426.43 W; Table 3), confirming mathematical reconstruction rather than predictive capability.

### 3.3. Anthropometric Constraints on Agility and Sprinting Phases

For change of direction ability (COD 5-0-5 total time), the algorithm retained adipose mass (Kerr) % as the sole non-zero feature:

Total Time COD-Timer 5-0-5 (s) = 4.8936 + 0.0035 × (Adipose Mass [Kerr], %)(3)

Worked Example (Arithmetic Reconstruction): For an athlete presenting the cohort mean adipose mass (27.61%), the reconstructed time is 4.8936 + (0.0035 × 27.61) = 4.99 s, which arithmetically reproduces the observed sample mean of 4.99 s.

For initial sprint acceleration (speed—0 to 5 m), the model retained a combination of lower-limb skinfolds, stature, and body composition fractions:

Speed—0 to 5 m (s) = 1.5920 − 0.0077 × (Height, cm) − 0.0089 × (Body Fat, %) + 0.0072 × (Muscle Mass, %) + 0.0010 × (Triceps Skinfold, mm) + 0.0048 × (Biceps Skinfold, mm) + 0.0007 × (Abdominal Skinfold, mm) + 0.0324 × (Thigh Skinfold, mm) + 0.0019 × (Hip Circumference, cm) + 0.0069 × (Bone Mass [Rocha], kg)(4)

Worked Example (Arithmetic Reconstruction): Inserting the cohort mean values for each retained predictor (height: 167.84 cm; body fat: 22.81%; muscle mass: 37.93%; triceps skinfold: 8.80 mm; biceps skinfold: 5.92 mm; abdominal skinfold: 18.25 mm; thigh skinfold: 11.93 mm; hip circumference: 89.02 cm; bone mass [Rocha]: 9.95 kg) yields:

1.5920 − (0.0077 × 167.84) − (0.0089 × 22.81) + (0.0072 × 37.93) + (0.0010 × 8.80) + (0.0048 × 5.92) + (0.0007 × 18.25) + (0.0324 × 11.93) + (0.0019 × 89.02) + (0.0069 × 9.95) = 1.0440 s, which corresponds exactly to the observed cohort sample mean of 1.0440 s (1.04 ± 0.12 s).

This calculation confirms the internal algebraic consistency in the unstandardization of regression coefficients. However, this arithmetic reconstruction should not be construed as evidence of out-of-sample predictive validity or generalizability.

### 3.4. Cross-Validation Performance and Resampling Stability Analysis

To evaluate out-of-sample predictive robustness and feature selection stability under small-sample constraints (n = 20), fivefold cross-validation and non-parametric bootstrap resampling (B = 500 replications) were conducted (Table 4). The regularized model for speed—0 to 5 m demonstrated moderate exploratory capacity (R^2^_cv_ = 59.42%, RMSE_cv_ = 0.08 s), with thigh skinfold (100.0% bootstrap selection), body fat % (99.4%), and muscle mass % (99.2%) identified as potentially relevant features. Here, it is important to clarify that the concept of bootstrap stability should be defined as the frequency with which the predictor is selected in the bootstrap samples and does not imply validated stability of the predictor or causal importance.

Under the conservative λ_1se_ penalty, regularized models for jump height CMJ (RMSE_cv_ = 5.99 cm), power CMJ (RMSE_cv_ = 432.30 W), COD 5-0-5 (RMSE_cv_ = 0.65 s), and total time 20 m (RMSE_cv_ = 0.36 s) yielded an R^2^_cv_ = 0.00%, indicating that their out-of-sample predictive capacity did not exceed that of the sample mean null model. Consequently, the non-zero coefficients retained in Equations (1)–(4) must be interpreted solely as sample-specific descriptive associations within this cohort. The arithmetic reconstruction shown in each worked example simply confirms that the algebraic transformation of coefficients was performed without error; true predictive validity was evaluated strictly through out-of-fold cross-validation (R^2^_cv_), which was moderate only for speed 0–5 m (R^2^_cv_ = 59.42%, RMSE_cv_ = 0.08 s).

## 4. Discussion

The objective of this study was to identify performance indicators based on body composition and physical fitness by profiling F5 players according to their playing roles. To this end, various stratified variables were analyzed for 20 players with disabilities using penalized regression models with ML. Based on this objective, the main findings showed that there are no differences among playing positions, which suggests that all players appear to have similar physical and anthropometric characteristics.

### 4.1. Body Composition Analysis and Physical Fitness

In the study led by Lameira Oliveira et al. [10], anthropometric variables were measured in 10 elite Brazilian teams, with average body fat percentages of 19.3%. Based on playing positions, center backs had an average of 23.1%, pivots had 21.5%, and wing players had the lowest values at 17.4%. In contrast to the results of the present study, they found higher values for attackers, with averages of 24.28 ± 5.16%, and lower values for defenders in the Colombian population, at 21.02 ± 7.48%, which were like those of the Brazilian centers. As for the Chilean population, as reported by Durán-Agüero et al. [33], they had an average of (~22.8%), like that of Colombian defenders.

A systematic review concluded that several scientific articles have evaluated and analyzed body composition in F5 players, reporting that, of the 10 studies included, the players’ body fat percentages ranged from 10.4% to 15.9% [11], with values lower than those found in the present study. Another anthropometric variable reported by Durán-Agüero et al. [33] was bone mass, with averages of 11.7 ± 1.1 kg and a bone mass percentage of 16.3 ± 1.2%; in comparison, the attackers in this study had a bone mass of 9.63 ± 1.01 kg and a percentage of 13.81 ± 1.87%, with averages lower than those of the defenders who reported an average bone mass of 10.34 ± 1.35 kg and a percentage of 15.22 ± 2.59%.

Furthermore, F5 is a sport that demands high levels of oxidative and glycolytic physical exertion due to the nature of the sport, which involves changes in direction, decelerations, technique, and the athlete’s ability to recover from fatigue in the shortest possible time [8]. Although in the results of this study no statistically significant positional differences were detected in performance variables including jumps (CMJ), changes of direction (COD), and speed, some previous studies that evaluated physical abilities such as power tests in a longitudinal study did report improvements in power, with average increases of 23.25% in minimum power, 11.08% in average power, and 3.5% in maximum power [8]. These discrepancies could be explained by methodological differences between the studies. In this study, as described in the results, no statistically significant differences were found between the anthropometric and physical fitness variables of defenders and attackers, as indicated by Student’s *t*-test and the Mann–Whitney U test.

When examining unadjusted bivariate relationships, some moderate-to-strong trends initially emerged in our cohort, such as the raw association between CMJ force and BMI (*ρ* = 0.606) or COD 5-0-5 time and sum of 8 skinfolds (*ρ* = 0.541). However, when controlling for the inflation of Type I errors using the Benjamini–Hochberg False Discovery Rate (FDR) correction, none of these bivariate correlations remained statistically significant (pFDR ≥ 0.05). This statistical reality represents a crucial finding: in small Paralympic cohorts (n = 20), traditional bivariate correlation matrices are highly susceptible to generating false-positive associations by sheer chance. Consequently, other weak raw trends, such as the relationship between muscle mass percentage and 20 m sprint time (r = 0.106), must be interpreted with extreme caution. This limitation directly underscores why relying on simple bivariate correlation coefficients to guide athletic preparation in visually impaired players can be misleading and strongly validates our decision to implement penalized multivariate algorithms (Lasso and Elastic Net) to parsimonious and exploratory factors.

### 4.2. Machine Learning in F5

Automatic ML models have attracted interest in sports science, such as the Elastic Net model and the Lasso algorithm, which identified and ranked anthropometric variables associated with the athletic performance of F5 players. Bongiovanni et al. [34] conducted an analysis of the contribution of anthropometric characteristics in professional soccer players using the Extra Trees Regressor (ETR) model and found that musculoskeletal structure such as muscle area, fat-free mass, and circumference are the main determinants of physical performance. In our exploratory models, cross-validated performance for jump and change-of-direction tasks was null (R^2^_cv_ = 0.00%); these associations remain hypothesis-generating.

Another study led by Izhanov et al. [35] conducted an analysis using an automated model, in which they found that performance is determined primarily by physical variables (20 m dribble, shooting, and dribbling) and only one anthropometric variable (total body mass), with technical-tactical variables predominating; in contrast, the model in this article assigns greater importance to anthropometric characteristics. However, Emmonds et al. [36] note that there is no relationship between changes in direction and adiposity variables in soccer players without disabilities.

### 4.3. Limitations and Future Prospects

This study highlights some methodological limitations when interpreting the results. First, the study was conducted at a single point in time; therefore, cross-sectional studies cannot provide a causal explanation of the phenomenon analyzed between anthropometric variables and physical fitness. For this reason, it is recommended that the findings of this study be interpreted with caution. Furthermore, the sample size is small and consists of participants at the same competitive level, which limits the generalizability of the data to other contexts.

Second, although the use of ML has made it possible to explore some potential trends in performance, validation in different contexts is needed to reinforce the results. In this regard, it is necessary to clearly acknowledge the limitations of the non-nested approach and the potential instability associated with the small sample size.

Third, penalized regression and bootstrap resampling (B = 500) mitigated overfitting and feature instability; however, the high ratio of parameters to samples (*p* = 32, n = 20) should be analyzed with caution, and this study should be considered preliminary. Consequently, the resulting equations should be interpreted with caution given the small sample size (n = 20). Thus, the results of the present study should be interpreted strictly as exploratory and hypothesis-generating, rather than as a validated predictive model. Future studies with larger cohorts are needed to confirm these preliminary associations.

Future lines of research should consider conducting longitudinal studies, analyses that incorporate other performance factors such as physiological, biochemical, technical, and tactical factors, and the implementation of studies that utilize ML [37]. Looking ahead, this study made it possible to rank anthropometric and physical performance variables using ML algorithms based on selection frequency in the models; these algorithms serve as useful tools for enhancing traditional statistics by revealing more complex patterns that are not typically utilized. Furthermore, the lack of specific parameters for playing positions affects the structure of physical training and talent identification; exploratory results suggest that anthropometric analysis could support training capacity and sports planning. More longitudinal studies, experimental research, and randomized controlled trials are needed to further explore these relationships in F5 and perhaps in other adapted sports such as seven-a-side soccer for people with cerebral palsy and soccer for amputees.

## 5. Conclusions

This study did not find any statistically significant differences in anthropometric profiles, body composition, or physical fitness between attackers and defenders in this national team cohort. Although no differences were observed among the sample of players evaluated in this study, ML algorithms suggested a preliminary set of anthropometric and physical fitness variables, where the characteristics retained in these specific equations describe descriptive associations specific to the sample rather than generalizable or robust determinants of performance. Future studies could validate the models used.

## Figures and Tables

**Table 1:** Basic anthropometric characteristics and structural morphology.

Variable	Attacker (n = 11)	Defender (n = 9)	Statistical Test	ES (95% CI)
Age (years)	30.73 ± 10.72	29.44 ± 10.31	Student’s *t*	0.12 (−0.76, 1.00)
Body Mass (kg) ^a^	71.20 [7.80]	69.20 [15.20]	Mann–Whitney U	0.15 (−0.35, 0.59)
Height (cm)	167.11 ± 4.77	168.73 ± 5.83	Student’s *t*	−0.31 (−1.19, 0.58)
BMI (kg/m^2^) ^a^	25.36 [2.30]	24.01 [4.93]	Mann–Whitney U	0.23 (−0.28, 0.64)
Arm Span (cm)	169.23 ± 9.77	170.04 ± 7.72	Student’s *t*	−0.09 (−0.97, 0.79)
Relaxed Arm Girth (cm)	27.46 ± 2.70	27.83 ± 4.76	Student’s *t*	−0.10 (−0.98, 0.78)
Flexed Arm Girth (cm)	28.71 ± 2.34	29.10 ± 3.74	Student’s *t*	−0.13 (−1.01, 0.76)
Waist Girth (cm)	79.75 ± 5.43	79.96 ± 12.65	Student’s *t*	−0.02 (−0.90, 0.86)
Hip Girth (cm) ^a^	87.50 [6.50]	87.50 [7.50]	Mann–Whitney U	−0.03 (−0.50, 0.45)
Mid-Thigh Girth (cm)	46.46 ± 5.42	48.42 ± 8.33	Student’s *t*	−0.29 (−1.17, 0.60)
Calf Girth (cm) ^b^	35.04 ± 2.24	35.49 ± 4.88	Welch’s t	−0.12 (−1.03, 0.79)
Humerus Diameter (cm)	6.13 ± 0.62	6.12 ± 0.81	Student’s *t*	0.01 (−0.87, 0.89)
Bi-styloid Diameter (cm)	5.43 ± 0.36	5.39 ± 0.34	Student’s *t*	0.11 (−0.77, 0.99)
Femur Diameter (cm)	8.43 ± 0.91	9.16 ± 1.06	Student’s *t*	−0.74 (−1.65, 0.18)
Waist-to-Hip Ratio	0.90 ± 0.04	0.88 ± 0.06	Student’s *t*	0.43 (−0.47, 1.31)
Waist-to-Height Ratio	0.48 ± 0.04	0.47 ± 0.07	Student’s *t*	0.07 (−0.81, 0.95)

Note. BMI: Body mass index; n: number; cm: centimeters; kg: kilograms; m: meters; CI: confidence interval; ES: effect size. Data are presented as mean ± standard deviation or median [interquartile range]. ^a^ Variable analyzed using the Mann–Whitney U test due to violation of normality assumptions. ^b^ Variable analyzed using Welch’s *t*-test due to heterogeneity of variances. ES (95% CI) represents effect size and its 95% confidence interval (Cohen’s *d* for parametric comparisons and rank-biserial correlation for non-parametric comparisons). No statistically significant differences were observed between attackers and defenders (*p* > 0.05).

**Table 2:** Body composition, skinfolds, and mass fractioning.

Variable	Attacker (n = 11)	Defender (n = 9)	Statistical Test	ES (95% CI)
Body Fat %	24.28 ± 5.16	21.02 ± 7.48	Student’s *t*	0.52 (−0.39, 1.41)
Muscle Mass %	37.53 ± 4.12	38.41 ± 5.23	Student’s *t*	−0.19 (−1.07, 0.70)
Triceps Skinfold (mm) ^a^	8.00 [3.50]	9.00 [2.50]	Mann–Whitney U	−0.11 (−0.56, 0.39)
Subscapular Skinfold (mm)	15.36 ± 6.96	16.50 ± 9.62	Student’s *t*	−0.14 (−1.02, 0.75)
Biceps Skinfold (mm)	6.41 ± 2.25	5.33 ± 1.66	Student’s *t*	0.54 (−0.37, 1.43)
Iliac Crest Skinfold (mm) ^a^	15.00 [11.25]	13.50 [12.40]	Mann–Whitney U	−0.07 (−0.53, 0.42)
Supraspinale Skinfold (mm) ^a^	16.50 [6.25]	15.00 [10.00]	Mann–Whitney U	0.07 (−0.42, 0.53)
Abdominal Skinfold (mm) ^a^	18.50 [14.50]	14.50 [27.50]	Mann–Whitney U	0.11 (−0.39, 0.56)
Front Thigh Skinfold (mm)	11.32 ± 2.71	12.67 ± 3.21	Student’s *t*	−0.46 (−1.35, 0.44)
Medial Calf Skinfold (mm)	8.96 ± 2.96	7.78 ± 3.36	Student’s *t*	0.38 (−0.52, 1.26)
Sum of 8 Skinfolds (mm)	105.31 ± 36.17	110.71 ± 57.42	Student’s *t*	−0.12 (−1.00, 0.77)
Sum of 6 Skinfolds (mm)	81.22 ± 27.13	86.22 ± 46.29	Student’s *t*	−0.14 (−1.02, 0.75)
Muscle Mass (Lee) %	38.39 ± 4.92	40.13 ± 5.33	Student’s *t*	−0.34 (−1.22, 0.55)
Muscle Mass (Lee) kg ^b^	26.81 ± 2.66	27.69 ± 5.76	Welch’s t	−0.19 (−1.10, 0.72)
Bone Mass (Rocha) %	13.81 ± 1.87	15.22 ± 2.59	Student’s *t*	−0.64 (−1.53, 0.28)
Bone Mass (Rocha) kg	9.63 ± 1.01	10.34 ± 1.35	Student’s *t*	−0.60 (−1.49, 0.31)
Residual Mass % ^a^	16.72 [15.89]	13.76 [7.10]	Mann–Whitney U	0.31 (−0.19, 0.69)
Residual Mass kg ^a^	11.39 [12.62]	8.02 [9.29]	Mann–Whitney U	0.39 (−0.10, 0.73)
Adipose Mass (Kerr) %	26.91 ± 6.17	28.46 ± 6.21	Student’s *t*	−0.25 (−1.13, 0.64)
Adipose Mass (Kerr) kg	18.83 ± 4.16	20.20 ± 8.06	Student’s *t*	−0.22 (−1.10, 0.67)
Fat Mass (Faulkner) %	15.11 ± 3.85	16.31 ± 6.20	Student’s *t*	−0.24 (−1.12, 0.65)
Fat Mass (Faulkner) kg ^a^	10.77 [3.62]	9.45 [6.34]	Mann–Whitney U	0.07 (−0.42, 0.53)
Fat Mass (Carter) %	11.12 ± 2.85	12.02 ± 4.64	Student’s *t*	−0.24 (−1.12, 0.65)
Fat Mass (Carter) kg ^a^	8.32 [2.39]	6.80 [4.59]	Mann–Whitney U	0.08 (−0.41, 0.54)

Note. %: percentage; mm: millimeters; kg: kilograms; CI: confidence interval; ES: effect size. Data are presented as mean ± standard deviation or median [interquartile range]. ^a^ Variable analyzed using the Mann–Whitney U test due to violation of normality assumptions. ^b^ Variable analyzed using Welch’s *t*-test due to heterogeneity of variances. ES (95% CI) represents effect size and its 95% confidence interval (Cohen’s *d* for parametric comparisons and rank-biserial correlation for non-parametric comparisons). No statistically significant differences were observed between attackers and defenders (*p* > 0.05).

**Table 3:** Lower limb power and dynamic capacities.

Variable	Attacker (n = 11)	Defender (n = 9)	Statistical Test	ES (95% CI)
Stride Length (cm)	102.25 ± 7.37	103.61 ± 8.50	Student’s *t*	−0.17 (−1.05, 0.71)
CMJ Jump Height (cm)	31.47 ± 6.28	32.35 ± 4.35	Student’s *t*	−0.16 (−1.04, 0.72)
CMJ Flight Time (ms)	497.11 ± 59.33	512.42 ± 34.30	Student’s *t*	−0.31 (−1.19, 0.58)
CMJ Force (N)	1478.57 ± 273.89	1345.24 ± 299.77	Student’s *t*	0.47 (−0.43, 1.35)
CMJ Velocity (m/s)	1.24 ± 0.14	1.26 ± 0.08	Student’s *t*	−0.17 (−1.05, 0.71)
CMJ Power (W)	1816.82 ± 481.17	1669.18 ± 359.13	Student’s *t*	0.34 (−0.55, 1.23)
COD 5-0-5 Total Time (s)	4.86 ± 0.59	5.15 ± 0.59	Student’s *t*	−0.51 (−1.40, 0.40)
COD 5-0-5 Average Speed (km/h)	7.56 ± 0.76	7.06 ± 0.82	Student’s *t*	0.62 (−0.29, 1.52)
COD Contact Time (ms)	772.13 ± 197.48	818.06 ± 215.35	Student’s *t*	−0.22 (−1.10, 0.66)
COD 5-0-5 10 m Time (s)	3.13 ± 0.45	2.89 ± 0.30	Student’s *t*	0.61 (−0.30, 1.50)
Sprint Time 0 to 5 m (s)	1.00 ± 0.12	1.09 ± 0.12	Student’s *t*	−0.75 (−1.66, 0.17)
Sprint Time 5 to 10 m (s)	0.78 ± 0.14	0.86 ± 0.08	Student’s *t*	−0.68 (−1.58, 0.23)
Sprint Time 10 to 15 m (s)	0.76 ± 0.14	0.78 ± 0.13	Student’s *t*	−0.13 (−1.01, 0.75)
Sprint Time 15 to 20 m (s) ^a^	0.89 ± 0.15	0.83 [0.12]	Mann–Whitney U	−0.04 (−0.51, 0.45)
Total Sprint Time 20 m (s) ^a^	3.30 [0.30]	3.56 [0.14]	Mann–Whitney U	−0.53 (−0.80, −0.06)

Note. cm: centimeters; ms: milliseconds; W: watts; km/h: kilometers per hour; s: seconds; m/s: meters per second; CI: confidence interval; ES: effect size. Data are presented as mean ± standard deviation or median [interquartile range]. ^a^ Variable analyzed using the Mann–Whitney U test due to violation of normality assumptions. ES (95% CI) represents effect size and its 95% confidence interval (Cohen’s *d* for parametric comparisons and rank-biserial correlation for non-parametric comparisons). No statistically significant differences were observed between attackers and defenders (*p* > 0.05).

**Table 4:** Cross-validated performance metrics and resampling feature stability (B = 500 bootstrap replications).

Dependent Var	Algorithm	α	λ_1se_	RMSE_cv_	R^2^_cv_	CPB (>5% Selection Frequency)
CMJ_h_ (cm)	Elastic Net	0.9	1.7422	5.99 cm	0.00%	Hip Circumference (36.8%), Thigh Skinfold (25.8%), Bone Mass Rocha % (25.8%), Age (23.6%)
CMJ_p_ (W)	Lasso (Top 5)	1	183.9785	432.30 W	0.00%	Body Mass (28.4%), Calf Circumference (23.2%), Bone Mass Rocha % (10.0%), BMI (9.4%)
COD-T (s)	Elastic Net	0.3	0.9666	0.65 s	0.00%	Adipose Mass Kerr % (27.8%), Iliac Crest Skinfold (25.8%), Subscapular Skinfold (24.4%)
Speed (s)	Elastic Net	0.1	0.0368	0.08 s	59.42%	Thigh Skinfold (100.0%), % Fat (99.4%), % Muscle (99.2%), Height (96.8%), Rocha % (95.4%)
TT (s)	Elastic Net	0.4	0.300	0.36 s	0.00%	Height (42.0%), Calf Skinfold (34.8%), Thigh Skinfold (23.6%), Lee % (16.6%)

Note: CPB = candidate predictors in bootstrap; CMJ_h_ = jump height; CMJ_p_ = power CMJ; COD-T = COD-timer 5-0-5 (s); speed = 0 to 5 m; TT = total time 20 m; Var: variable.

## Data Availability

The original findings presented in this study (data on anthropometric and physical fitness metrics of the athletes evaluated) are included in the article. The following supporting information can be displayed at https://doi.org/10.6084/m9.figshare.33260565 (accessed on 14 August 2026).
